# Thermo-Hydrodynamic Lubricating Behaviors of Upstream Liquid Face Seals with Ellipse Dimples

**DOI:** 10.3390/ma16083248

**Published:** 2023-04-20

**Authors:** Shaoxian Bai, Kaixin Li, Jing Yang, Shiyi Bao, Chunhong Ma

**Affiliations:** 1 College of Mechanical Engineering, Zhejiang University of Technology, Hangzhou 310032, China; 2 School of Mechanical and Energy Engineering, Zhejiang University of Science and Technology, Hangzhou 310023, China

**Keywords:** thermo-hydrodynamic liquid behaviors, face seal, ellipse dimples, upstream pumping, cavitation effect

## Abstract

In order to obtain the leakage characteristics of an upstream pumping face seal with inclined ellipse dimples under high-temperature and high-speed liquid lubricating conditions, a thermo-hydrodynamic lubricating model is developed. The novelty of this model is that it takes the thermo-viscosity effect and cavitation effect into account. The influence of operating parameters, such as rotational speed, seal clearance, seal pressure, ambient temperature and structural parameters, such as dimple depth, inclination angle, slender ratio and dimple number on the opening force and leakage rate, is numerically calculated. The results obtained show that the thermo-viscosity effect makes the cavitation intensity decrease noticeably, leading to an increase in the upstream pumping effect of ellipse dimples. Moreover, the thermo-viscosity effect may make both the upstream pumping leakage rate and opening force increase by about 10%. It can also be found that the inclined ellipse dimples can produce an obvious upstream pumping effect and hydrodynamic effect. Based on the reasonable design of the dimple parameter, not only can the sealed medium achieve zero leakage, but the opening force can also increase by more than 50%. The proposed model has the potential to provide the theoretical basis for and guide the future designs of upstreaming liquid face seals.

## 1. Introduction

The upstream pumping design of mechanical face seals is an efficient way to reduce sealing leakage and contact wear. Muti-dimple face seals have proven efficient in wear reduction through careful design [1,2,3,4]. Meanwhile, directional dimples, such as ellipse shapes, may produce significant upstream pumping effects, providing more potential design approaches for longer service life and low-leakage seals [5,6,7]. However, the upstream pumping effect and the effective control of the leakage rate depend on the careful design of face grooves and are affected by many factors. Among these factors, the fluid cavitation in the groove area not only affects the opening force but also affects the leakage rate. Moreover, with the continuous increase in equipment working speed, the decrease of fluid viscosity caused by the temperature rise leads to the variation of cavitation area, which also affects the upstream pumping effect and causes the thermal cavitation problem. Therefore, the novelty of this study is to propose a comprehensive model to investigate the thermo-hydrodynamic lubricating behavior, taking the fluid cavitation effect and thermos-viscosity effect into consideration, which is more reasonable and urgent for obtaining the leakage characteristics of upstream pumping face seals.

Over the past decades, published works have shown that geometric grooves with upstream pumping capabilities were feasible for achieving a zero-leakage design of the sealed medium [8,9,10,11,12,13,14]. In 1984, Etsion [8] pointed out that the effective design of grooves on the sealing surface might contribute to achieving zero leakage. In 1994, based on the pumping principle of a spiral groove, Lai et al. [15] confirmed through experiments that the upstream pumping mechanical seal of a spiral groove could achieve non-contact sealing and “zero” leakage. In 2008, Lebeck ‘s work [16] showed that “gas seal oil” between low pressure air and high pressure oil could achieve zero leakage with the design of upstream pumping linear grooves, and the experiment confirmed that the feasibility of the mutual sealing of the gas phase and liquid phase fluid. The theoretical analysis [7,15,16,17] proved that the inclined elliptical dimples could not only present enough upstream pumping effect to realize the complete reverse leakage of sealed medium, but also generate obvious dynamic pressure effect to keep the non-contact operation of sealing faces, which could provide another feasible way for the design of upstream pumping sealing faces.

Based on numerous theoretical works about the upstream pumping effect of face seals, it was found that this effect was largely depends on the shear speed and fluid viscosity of the sealing medium [18,19,20]. Meanwhile, the upstream pumping effect is significantly affected by cavitation [21,22,23,24,25]. In experiments, Nau [26] observed the occurrence of cavitation in mechanical seals with tap-water or mineral oil at a speed of 530 rpm and summarized that the leakage rate relied on the circumferential extent of the cavitation, decreasing as it expanded. Li’s work [27] on spiral groove oil film seals also showed that the cavitation could reduce film thickness and leakage. It was shown that at the speed of 2000 rpm and the spiral angle of 22.5°, the film thickness decreased by about 31.5% when the pressure enhanced fourfold from 0.2 MPa, which also experimentally indicated the decrease in change of leakage rate.

Moreover, for the high-speed upstream pumping face seals, when the thermo-viscosity effect was considered, change of both the shear ratio and cavitation intensity would make the upstream pumping effect more complex, since these two factors were coupled together [28,29,30]. Relatedly, Zhang and Meng’s experimental work on thrust bearings [28] reported that the cavitation originated at the downstream side of the grooves and expanded towards the upstream side with increasing speed. The increasing rotational speed might increase cavitation intensity. On the contrary, for the pocket pivoted-pad thrust bearing with diameter 171.45 mm, the lubricating oil film might present an increasing temperature with the increase of rotational speed, reaching about 15 °C at the rotational speed of 3000 rpm [29]. The increasing temperature might lead to lower viscosity, and then cavitation intensity became weaker.

Therefore, this paper aims to propose a modified thermo-hydrodynamic model to investigate the lubricating behavior of face seals with elliptical dimples. This model not only takes the thermo-viscosity effect and cavitation effect into consideration but also obtain the simulating results under high-temperature and high-speed liquid lubricating conditions. The proposed model has the potential to provide the theoretical basis and design guidance for the upstreaming liquid face seals.

## 2. Theoretical Model

### 2.1. Geometric Model

**Figure 1:**
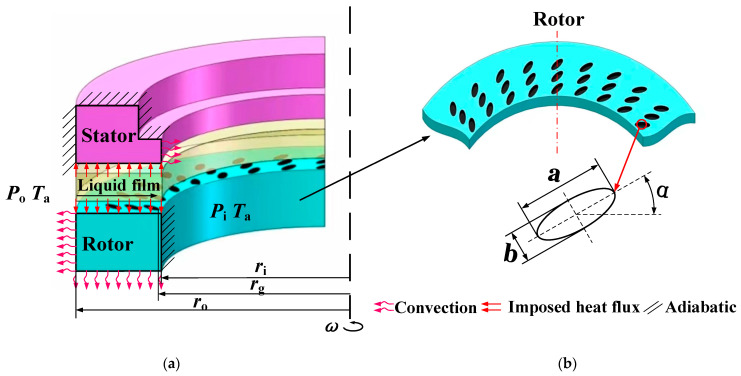
Schematic diagram of porous end face seal structure and boundary conditions. (**a**) sealing structure, (**b**) surface groove and boundary condition.

The theoretical model of an upstream pumping face seal with ellipse dimples is shown in Figure 1. Evenly distributed elliptical dimples are machined on the rotor face, where *r*_i_ and *r*_o_ are the inner and outer radii of the sealing ring, and *r*_g_ is the starting radius of the groove on the end face of moving ring. The specific structural parameters of surface grooves (dimples) are listed in Table 1. In this study, in order to analyze the heat transfer situation between ambient and solid, the thermal boundary conditions were set as convective, adiabatic and imposed heat on the surface, as shown in Figure 1.

In order to simplify the simulation process, the periodic region was selected as the simulating domain. The mesh density of this domain is 60 × 60 × 33, which is enough to satisfy the simulation accuracy. The materials of moving ring and static ring are stainless steel and graphite, respectively. Their detailed parameters for this calculation are shown in Table 2. The lubricant parameters used in this study are shown in Table 3.

### 2.2. Governing Equations

Fluid lubrication based on the Reynolds equation has been widely applied in the numerical analysis of face seals. For thermohydrodynamic lubrication analysis of face seals, the mathematical model mainly includes the Reynolds equation, energy equation, solid heat conduction equation [24] and cavitation equation [19,25].

Considering the effect of surface roughness, Reynolds equation for oil film lubrication with the introduction of the PC average flow model and the contact factor proposed by Wu et al. [31] is:

(1) $$\frac{\partial }{r\partial \theta }\left(Q_{\theta }\frac{\rho h^{3}}{\eta }\frac{\partial p}{r\partial \theta }\right)+\frac{\partial }{r\partial \theta }\left(Q_{r}\frac{r\rho h^{3}}{\eta }\frac{\partial p}{\partial r}\right)=6\omega Q_{s}\frac{\partial \rho h}{\partial \theta }$$

where *h* is the thickness of the lubricating film, *p* is the pressure of the lubricating film, *ω* is the rotating speed of the rotor, *ρ* is the density of the lubricating medium, *r* and *θ* are the radials and circumferential coordinates of the selected calculation area, *η* is the viscosity of the lubricating medium, *Q_θ_* is the circumferential pressure flow factor, *Q_r_* is the radial pressure flow factor, *Q_s_* is the shear flow factor.

As discussed in works [19,25], in the cavitation region, the compressible of fluid (the density variation) is considered, and the pressure distribution in the cavitation region can be solved. The following density equation can be used to describe the cavitation effect in the liquid lubrication analysis.

(2) $$\rho =\left\{ \begin{array}{l} \frac{\rho_{0}}{p_{c}}p\;\;\;\;\mathrm{if}\;\;\;\;p\leq p_{c}\;\;\\ \rho_{0}\;\;\;\;\;\;\mathrm{if}\;\;\;\;p>p_{c}\; \end{array}\right.$$

where, *p_c_* is cavitation pressure, and *ρ*_0_ is the fluid density at liquid state. Based on the experimental value, cavitation pressure in this study is set as 0.03 MPa [28].

When considering the influence of viscosity by temperature, the viscosity equation of lubricating oil is given:

(3) $$\eta =\eta_{0}\exp\left[-\beta \left(T-T_{0}\right)\right]$$

where *β* is the corresponding temperature-viscosity coefficient, *η*_0_ is the initial viscosity of the lubricating oil, *T*_0_ is the initial temperature of the lubricating oil. Here, *β* = 0.031 K^−1^.

Considering the heat of contact friction on the solid surface, the energy equation of the sealing film can be modified to the following form:

(4) $$\begin{array}{l} \left(Q_{s}\frac{\omega rh}{2}-Q_{\theta }\frac{h^{3}}{12\eta }\frac{\partial p}{r\partial \theta }\right)\frac{\partial T}{r\partial \theta }-\frac{\partial }{\partial r}\left(Q_{r}\frac{h^{3}}{12\eta }\frac{\partial p}{\partial r}\right)\frac{\partial T}{\partial r}\\ =\frac{\eta \omega^{2}r^{2}}{h\rho c_{v}}-\frac{h^{3}}{12\eta \rho c_{v}}\left[{\left(\frac{\partial p}{r\partial \theta }\right)}^{2}+{\left(\frac{\partial p}{\partial r}\right)}^{2}\right]+\frac{fp_{c}\omega r}{\rho c_{v}}\\ +\frac{k_{g,s1}}{\rho c_{v}}\left(T_{s1}-T\right)+\frac{k_{g,s2}}{\rho c_{v}}\left(T_{s2}-T\right) \end{array}$$

where, *p*_c_ is the bearing capacity per unit area of the contact surface rough peak [32], *T*_s_ is the static ring temperature, *c*_v_ is the specific heat capacity at constant volume, *k*_g,s1_ and *k*_g,s2_ are the thermal convective heat transfer coefficients at the interface of relative motion, respectively, *T*_s1_ and *T*_s2_ are the solid surface temperatures at the interface of relative motion.

(5) $$p_{c}=\frac{16\sqrt{2}}{15}\pi {\left(\mu \alpha \sigma \right)}^{2}\sqrt{\frac{\sigma }{\alpha }}EF_{2.5}(\lambda )$$

(6) $$\frac{1}{E}=\frac{1}{2}\left(\frac{1-v_{1}^{2}}{E_{1}}+\frac{1-v_{2}^{2}}{E_{2}}\right)$$

The calculation in this paper does not consider the influence of surface waviness for the time being, and assuming that the height of surface roughness peak conforms to the Gaussian distribution law, the formula for *F*_2.5_(*λ*) is

(7) $$F_{2.5}(\lambda )=\frac{1}{\sqrt{2\pi }}\int_{\lambda }^{\infty }{\left(s-\lambda \right)}^{2.5}e^{-\frac{s^{2}}{2}}ds$$

where, *μ*, *α*, *σ* are roughness characterization parameters, taking the value range of *μασ* from 0.04 to 0.08. *λ* is the film thickness ratio, *λ* = *h*/*σ. E* is the integrated elastic modulus. *E*_1_ and *E*_2_ are the elastic modulus of the two contact surfaces, respectively. *v*_1_ and *v*_2_ are Poisson’s ratios.

The temperature of the sealing ring is usually calculated by the heat conduction equation, and the heat conduction equation of the moving ring is

(8) $$\frac{\partial^{2}T_{s}}{r^{2}\partial \theta^{2}}+\frac{\partial }{r\partial r}\left(r\frac{\partial T_{s}}{\partial r}\right)+\frac{\partial^{2}T_{s}}{\partial z^{2}}=0$$

The static heat conduction equation is

(9) $$\frac{k_{c2}}{\rho_{s2}c_{s2}}[\frac{\partial^{2}T_{s}}{r^{2}\partial \theta^{2}}+\frac{1}{r}\frac{\partial }{\partial r}(r\frac{\partial T_{s}}{\partial r})+\frac{\partial^{2}T_{s}}{\partial z^{2}}]=\omega \frac{\partial T_{s}}{\partial \theta }$$

where *T_s_* is the static ring temperature, *k*_c2_ is the thermal conductivity of the static ring material. *ρ_s_*_2_, *c_s_*_2_ are the corresponding density and specific heat capacity.

The following pressure boundary conditions are applied:

(10) $$p(r=r_{i},\theta )=p_{i}$$

(11) $$p(r=r_{o},\theta )=p_{o}$$

(12) p(r,θ=π/Z)=p(r,θ=−π/Z)

The axial dynamic temperature boundary conditions are

(13) $$\mathrm{if}\;q_{r}\left(\theta ,r=r_{i}\right)<0,\;T\left(\theta ,r=r_{i}\right)=T_{\mathrm{in}}$$

(14) $$\mathrm{if}\;q_{r}\left(\theta ,r=r_{o}\right)>0,\;T\left(\theta ,r=r_{o}\right)=T_{\mathrm{out}}$$

The parameters to measure the sealing performance mainly include the opening force *F*_0_ and the leakage rate *Q*. The dimensionless calculation formulas are:

(15) $$F_{0}=\frac{1}{p_{a}r_{}^{2}}\int_{0}^{2\pi }\int_{r_{i}}^{r_{o}}prdrd\theta$$

(16) $$Q=\frac{1}{h^{3}p_{a}}\int_{0}^{2\pi }h^{3}r\frac{\partial p}{\partial r}d\theta$$

### 2.3. Model Validation

**Figure 2:**
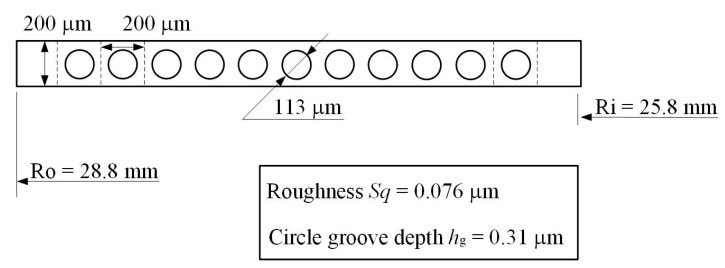
Textured seal ring with circle-dimples.

In order to validate the model, the temperature rise between the experimental work on aqueous multi-circle-dimpled face seal by Dingui [3] and this proposed model were compared. The structure parameters of the seal are illustrated in Figure 2.

**Figure 3:**
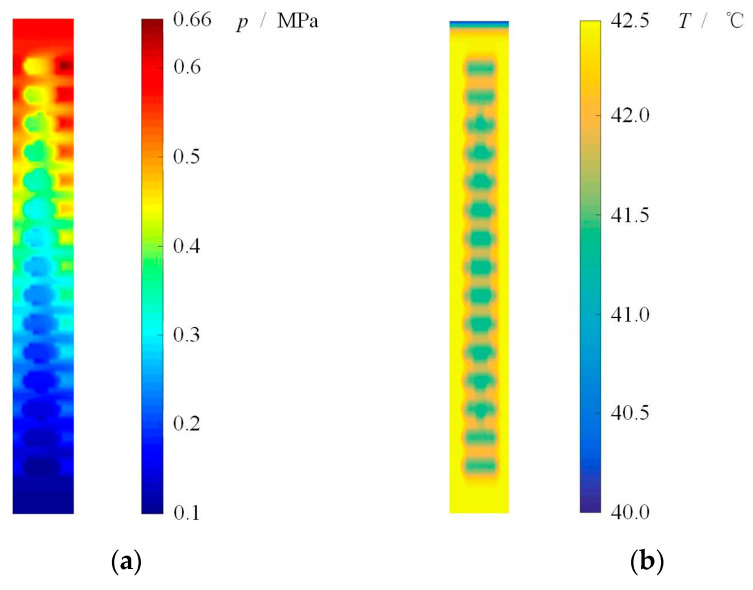
Pressure and temperature distributions of sealing film. (*ω* = 1000 rpm, *p*_o_ = 0.6 MPa, *p*_i_ = 0.1 MPa). (**a**) Pressure distribution, (**b**) Temperature distribution.

**Figure 4:**
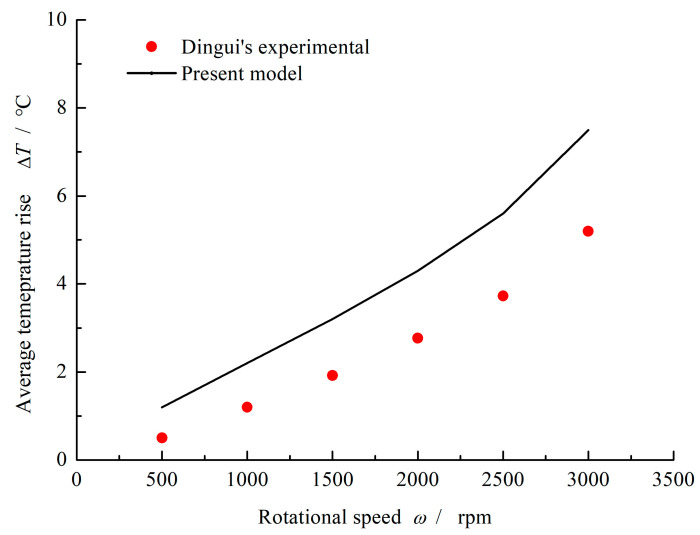
Comparison of numerical results and experimental results. (*p*_o_ = 0.6 MPa, *p*_i_ = 0.1 MPa).

The pressure and temperature distributions obtained by present model are shown in Figure 3. Obviously, under aqueous lubrication condition, the multi-circle-dimpled face present hydrodynamic effect and film temperature rise. Here, when the rotational speed *ω* = 1000 rpm, sealing pressure *p*_o_ = 0.6 MPa and ambient pressure *p*_i_ = 0.1 MPa, the maximum pressure of 0.66 MPa as well as the maximum temperature rise of 2.5 °C is produced. With the increase of rotational speed, the film temperature increases accordingly. The average film temperature rise calculated by the present model is compared with the Dingui’s experimental, as shown in Figure 4. It can be seen that the average film temperature rise increases from about 1.2 °C to 7.5 °C with increasing rotational speed from 500 rpm to 3000 rpm. The temperature rise obtained by the current model is always little higher than the literature experimental at different speed. Meanwhile, the error of temperature rise between the literature and the current model keeps stable, about 30~40%. The more important is that the current model gives the same trend of temperature change as mentioned in the literature.

## 3. Results and Discussion

**Figure 5:**
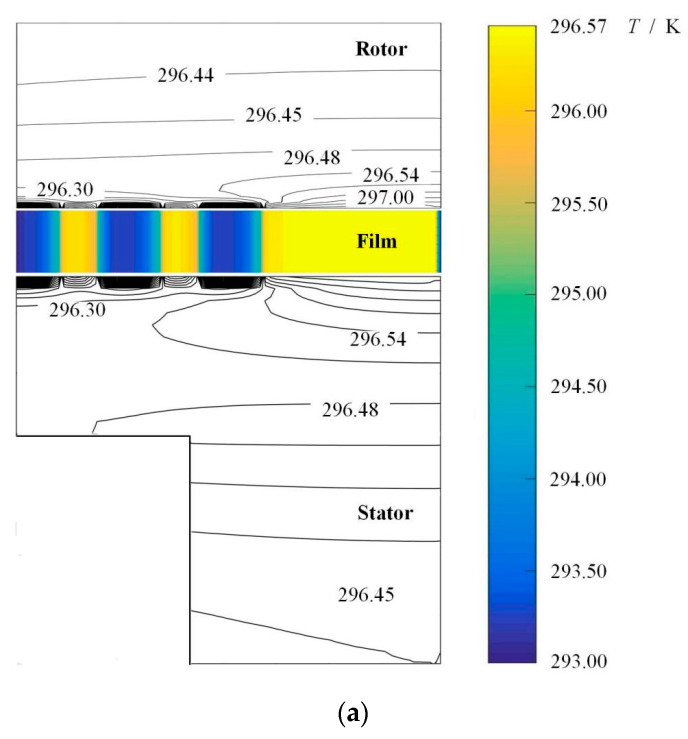

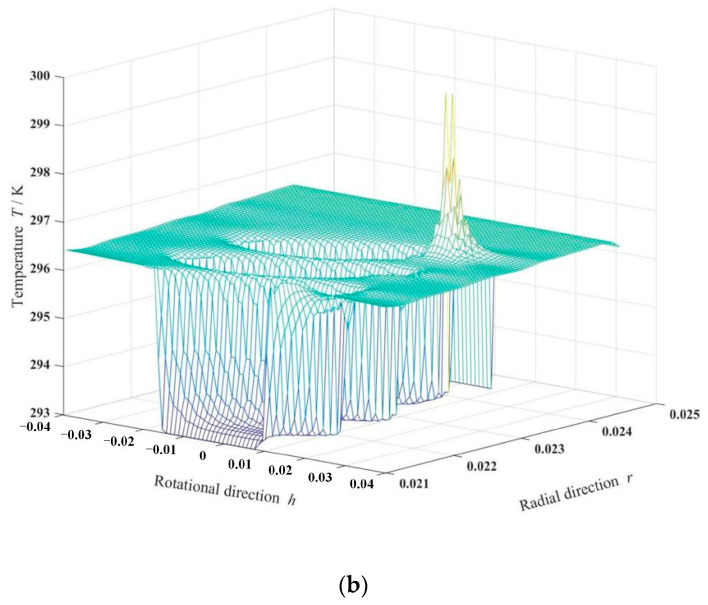
Cross-sectional temperature fields of seal and temperature isogram of sealing film. (*ω* = 20,000 rpm, *P*_o_ = 4, *h*_0_ = 2 μm, *h*_d_ = 5 μm, *b* = 0.20 mm, *γ* = 3, *α* = 45°, *N* = 80). (**a**) Cross-sectional temperature fields of seal, (**b**) Temperature isogram of sealing film.

There is a temperature difference of 3.5 K from the outlet to inlet of the oil film between the seal faces as shown in Figure 5, and the temperature difference between the seal rings is about 0.7 K. The reason for this phenomenon is that the oil is pumped by the ellipse dimples from low pressure into the clearance at inner radius, and the different temperature distributions in the cross sections of the stator and the rotor are owing to the difference in thermal conductivity.

It can also be seen that the temperature of the oil film gradually increases from the inside radius to the outside radius due to the shear action of speed. It is clear that the temperature of the sealing medium is lowest at the entrance of the dimples at the low pressure inside radius and gradually increases from inside radius to outside radius. The temperature of the sealing medium reaches a maximum of 298.5 K at the dimple root near outside radius. The reason for this temperature distribution is that the ellipse dimples present obvious upstream pumping effect, and the oil is pumped to continuously flow from inside radius to outside radius, speed shear leading to a temperature rise.

**Figure 6:**
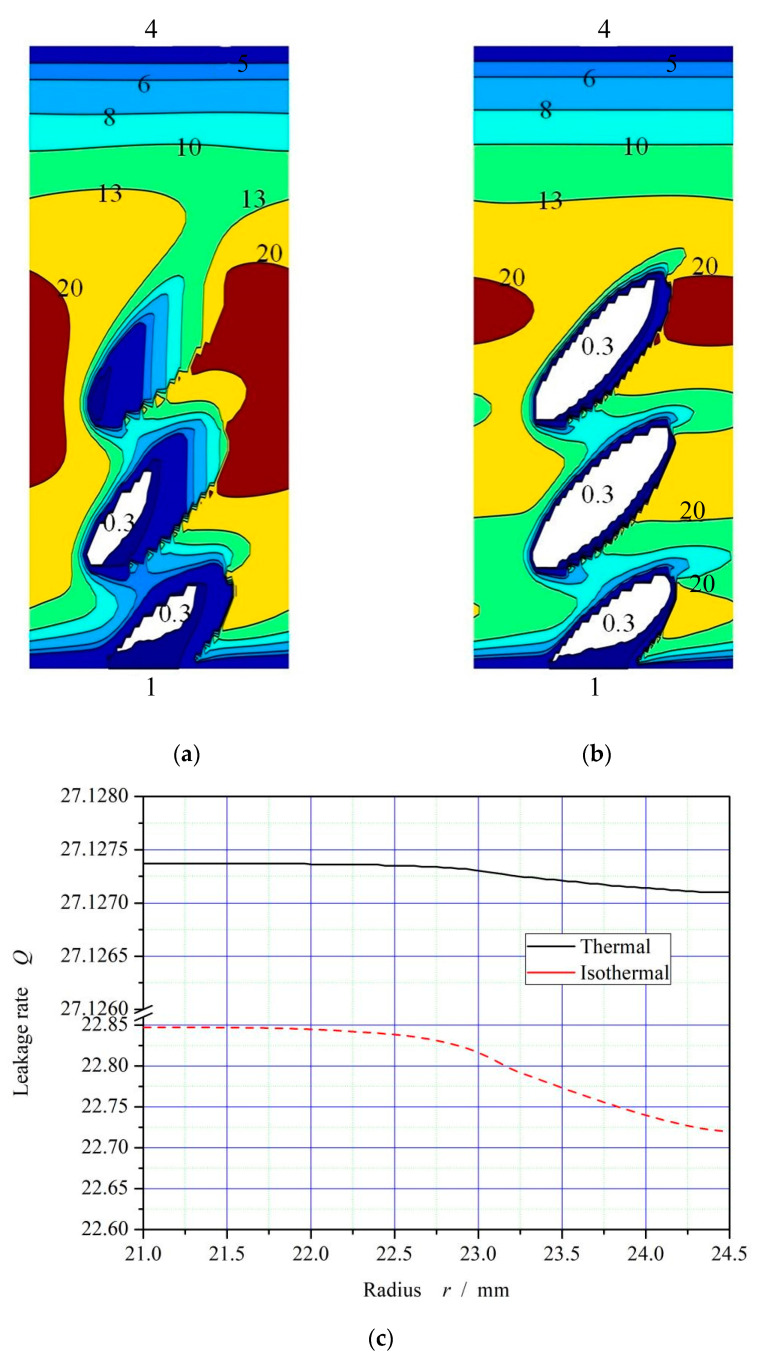
Schematic diagram of the pressure distributions and flow conservation (*ω* = 20,000 rpm, *P*_o_ = 4, *h*_0_ = 2 μm, *h*_d_ = 5 μm, *b* = 0.20 mm, *γ* = 3, *α* = 45°, *N* = 80). (**a**) Thermohydrodynamic pressure distribution; (**b**) Isothermal pressure distribution; (**c**) Curves of flow conservation.

The increase of temperature leads to the decrease of oil film viscosity, which will theoretically lead to the weakening of cavitation effect. As shown in Figure 6, the cavitation area in the dimple area, with pressure lower than 0.3 MPa, decreases significantly and the pressure distribution changes obviously after considering the temperature viscosity effect of the oil. The decrease of viscosity results in the complete disappearance of cavitation in the third dimple near the high-pressure side.

The leakage of sealing fluid from the high-pressure side to the low-pressure side is defined as positive leakage, and vice versa. As can be seen from Figure 6a,b, under the shear rate, the ellipse dimples generate upstream pumping to the high-pressure side, and forms a circumferential high pressure isoline of 1.0 Mpa under both thermohydrodynamic and isothermohydrodynamic conditions, higher than the outer sealing pressure of 0.4 Mpa in the high-pressure area, indicating that the ellipse-dimple face can achieve complete reverse leakage. As shown in Figure 6c, the leakage rate presents a positive value.

It should be also noted that, the leakage rate is essentially the radial flow rate, and the accuracy of the leakage rate analysis is improved through the conservation of flow rate in the calculation process. The radial flow deviation is less than 1% according to this figure. When the thermal viscosity effect is taken into account, the leakage rate increases from about 22.8 to about 27.1. The reason may be that the thermo-viscosity effect results in an obvious increase of upstream pumping effect.

The upstream pumping effect of elliptical microporous end face seals is affected not only by the texture geometry, but also by the working parameters. Next, the leakage behavior of end face seals with elliptic microporous texture under different working parameters and geometric structures will be further discussed when considering the thermal cavitation effect.

### 3.1. Operating Conditions

#### 3.1.1. Rotational Speed

**Figure 7:**
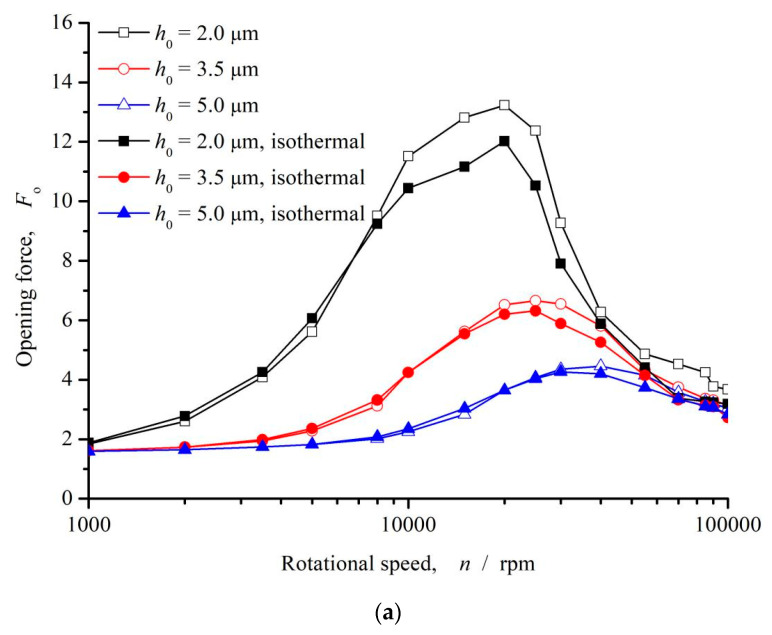

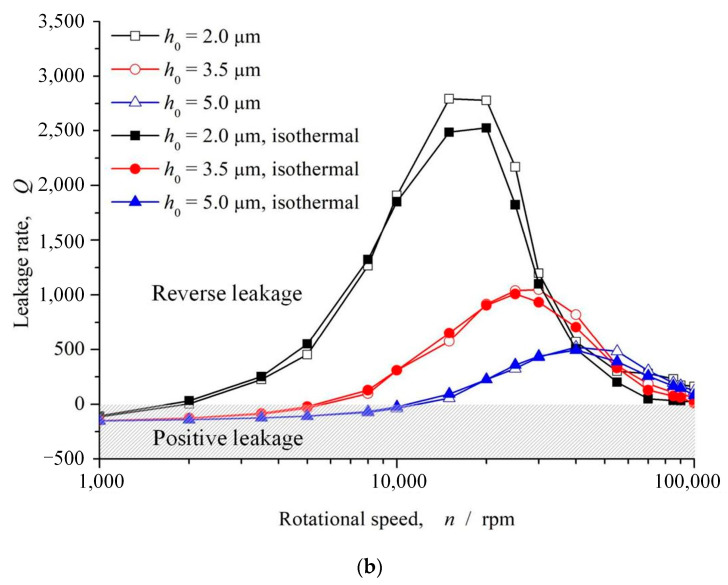
Influence of rotational speed on opening force and leakage rate (*P*_o_ = 4, *h*_0_ = 2 μm, *h*_d_ = 5 μm, *b* = 0.20 mm, *γ* = 3, *α* = 45°, *N* = 80). (**a**) Opening force; (**b**) Leakage rate.

Figure 7 shows the influence of rotational speed on opening force and leakage rate. As can be seen that, with the increase of speed, the opening force presents a trend of increasing first and then decreasing. When the speed is between 10,000 and 30,000 rpm, the opening force reaches its maximum value. When the speed exceeds 30,000 rpm, the opening force gradually decreases and becomes stable with the continuous increase of speed. However, as a whole, the hydrodynamic pressure effect caused by shear action increases the opening force, which can increase by much more than 100%. With the increase of rotational speed, the leakage rate rapidly transforms from negative to positive, that is to say, from positive leakage to reverse leakage, forming the complete upstream pumping of the fluid. When the rotational speed exceeds 10,000 rpm, the leakage rate is basically transformed into reverse pumping, and with the continuous increase of the rotational speed, the reverse pumping is continuously enhanced. When the rotational speed reaches 20,000 rpm, the reverse pumping effect is the strongest, and then with the continuous increase of the rotational speed, the reverse pumping effect is constantly weakened. In addition, when the rotational speed exceeds 100,000 rpm, the leakage rate tends to 0, and there is a chance to realize the phenomenon of zero leakage of the sealed end face.

The opening force increases and then decreases with the rotational speed, which is mainly due to the effect of cavitation in the microdimple region on the upstream pumping of microdimple. The upstream pumping effect of the microdimple is mainly due to the liquid entering the dimple area under the action of velocity shear. Due to the existence of the deep dimple, the fluid flow resistance is small, which makes part of the liquid flow along the long axis of the microdimple, forming the upstream pumping from the low-pressure side to the high-pressure side. Meanwhile, the extrusion of the fluid forms the dynamic pressure effect, which increases the opening force. On the other hand, when the liquid enters the dimple zone along the circumferential direction under the action of shear velocity, the pressure suddenly decreases due to the divergence of flow space. When the cavitation pressure reaches the cavitation zone, the cavitation zone is formed in the dimple zone, which obstructs the flow of the fluid along the radial direction, thus weakening the upstream pumping effect. With the increase of the velocity, the cavitation intensity increases, and the influence on the upstream pumping gradually increases. As a result, opening force and leakage rate appear inflection points in the range of 10,000~30,000 rpm.

It should be noted that as the viscosity of the fluid decreases with increasing rotational speed and temperature, the upstream pumping capacity increases as well as the opening force as theoretically expected, about 10% in degree at rotational speed 20,000 rpm. This is due to the decrease of cavitation intensity, resulting in the increase of fluid film loading capacity, which is consistent with the literature analysis [27].

#### 3.1.2. Seal Clearance

**Figure 8:**
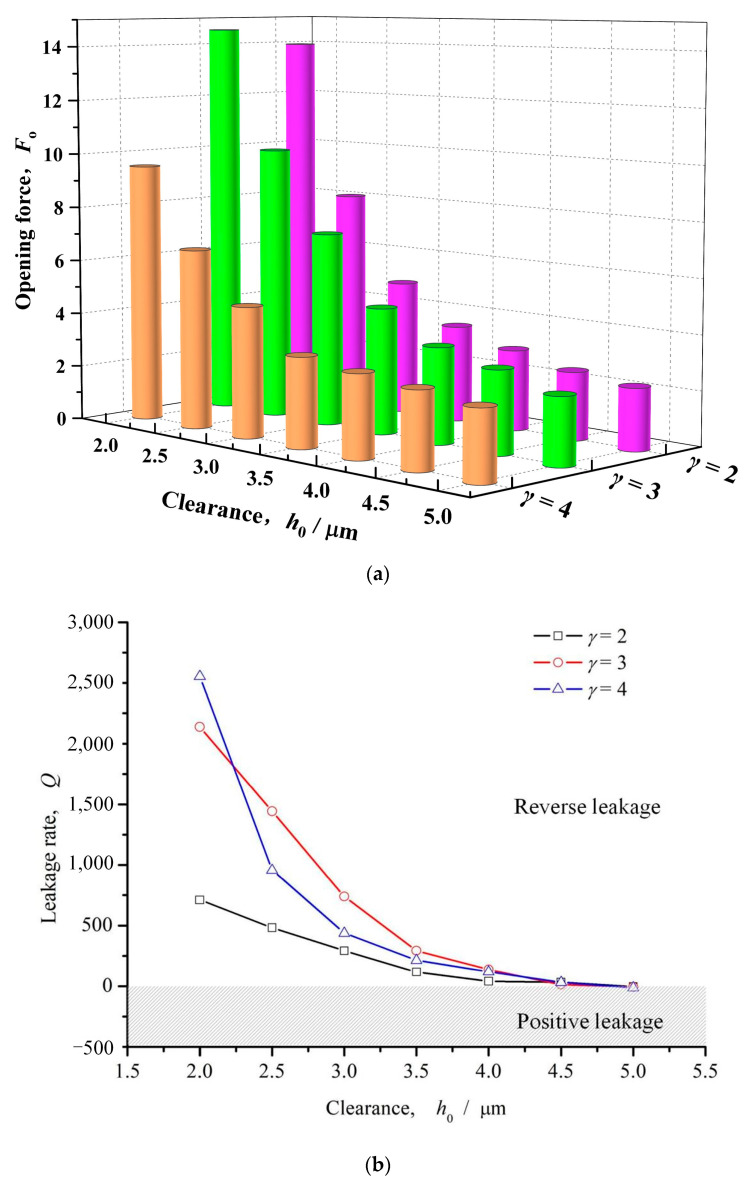
Influence of seal clearance on opening force and leakage rate (*ω* = 12,000 rpm, *P*_o_ = 4, *h*_d_ = 5 μm, *b* = 0.20 mm, *γ* = 3, *α* = 40°, *N* = 80). (**a**) Opening force; (**b**) Leakage rate.

Figure 8 shows the influence of seal clearance on opening force and leakage rate. It can be seen that with the increase of sealing clearance, the opening force and upstream pumping leakage rate with different dimple slender ratio present similar trend, decreasing quickly with the increase of clearance. When the slender ratio *γ* = 2, the opening force decreases larger than 80% with the increase of the seal clearance from 2 μm to 5 μm, while the leakage rate transfers from positive value to negative value at clearance 5 μm.

As described above, cavitation effect impedes upstream pumping of ellipse dimples. With the increase of the seal clearance, the shear rate decreases, and the pumping effect of velocity shear on the fluid along the long axis of the elliptical microdimple decreases. Meanwhile, the cavitation effect decreases the movement resistance of the fluid in the dimple area, which is conducive to the upstream pumping flow of the liquid.

#### 3.1.3. Seal Pressure

**Figure 9:**
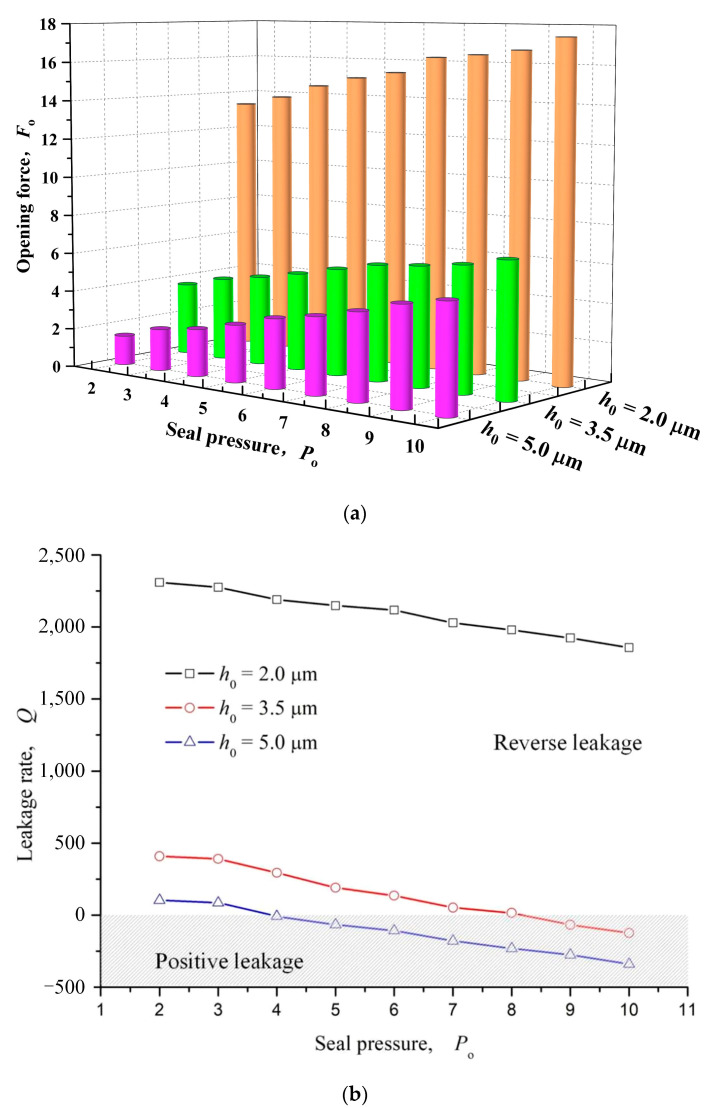
Influence of seal pressure on opening force and leakage rate (*ω* = 12,000 rpm, *h*_0_ = 2 μm, *h*_d_ = 5 μm, *γ* = 3, *α* = 40°, *N* = 80). (**a**) Opening force; (**b**) Leakage rate.

Figure 9 shows the influence of seal pressure on opening force and leakage rate. As can be seen that, with the increase of seal pressure, opening force and leakage rate both show a monotonous trend, that is, opening force linearly increases with the increase of sealing pressure, leakage rate gradually decreases from positive to negative with the increase of sealing pressure, and the reverse pumping changes to forward pumping. The main reason for this change is that with the increase of sealing pressure, the sealing fluid film pressure increases, the opening force increases, and the positive pressure flow of the fluid is enhanced, which turns into positive leakage when it exceeds the upstream pumping.

#### 3.1.4. Seal Temperature

**Figure 10:**
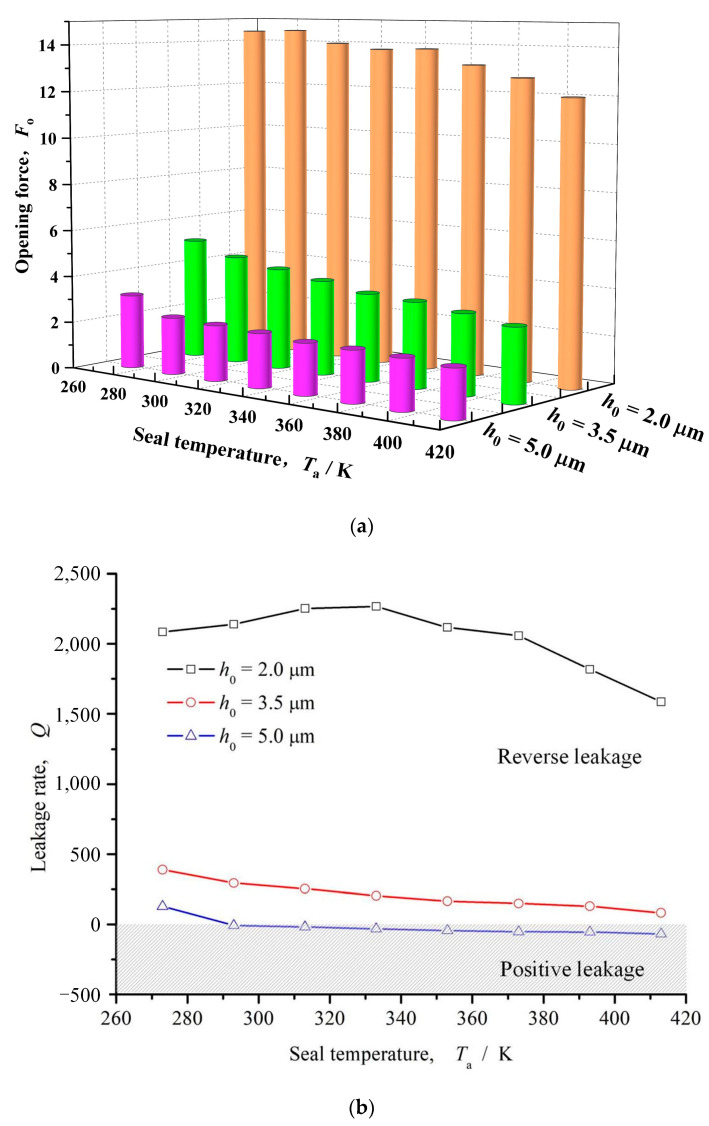
Influence of seal temperature on opening force and leakage rate (*ω* = 12,000 rpm, *P*_o_ = 4, *h*_0_ = 2 μm, *h*_d_ = 5 μm, *γ* = 3, *α* = 45°, *N* = 80). (**a**) Opening force; (**b**) Leakage rate.

Figure 10 shows the influence curve of sealing temperature on opening force and leakage rate. It can be seen from this figure that with the increase of sealing temperature, the opening force and leakage rate show a downward trend as a whole, and the leakage rate gradually decreases from positive to negative with the increase of sealing temperature, and the reverse pumping changes to forward pumping.

### 3.2. Geometric Configuration

#### 3.2.1. Dimple Number

**Figure 11:**
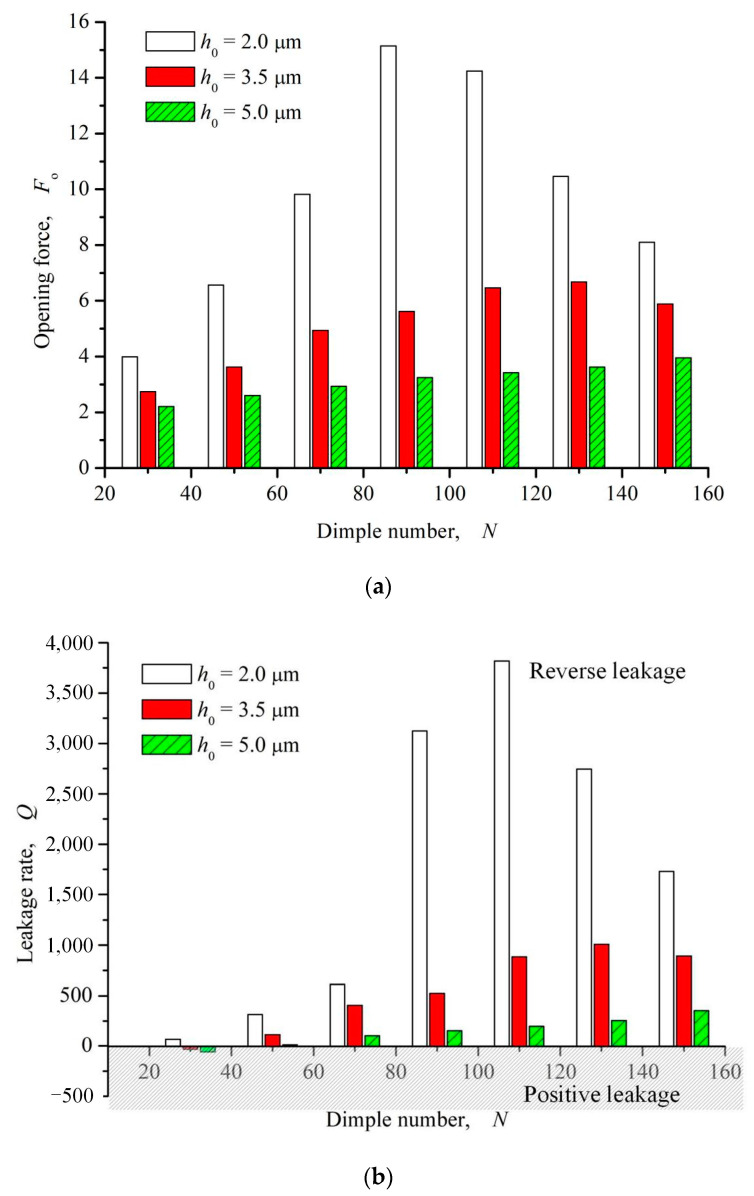
Influence of dimple number on opening force and leakage rate (*ω* = 12,000 rpm, *P*_o_ = 4, *h*_0_ = 2 μm, *γ* = 3, *α* = 45°). (**a**) Opening force; (**b**) Leakage rate.

Figure 11 shows the influence curve of circumferential dimple number on opening force and leakage rate. It can be seen from the figure that with the increase of the number of circumferential microdimples, the opening force on the whole presents a trend of first increasing and then decreasing. With the increase of opening force, the upstream pumping capacity of elliptical microdimples is continuously enhanced. When the number of circumferential dimples is larger than 30, the leakage rate gradually changes from positive leakage to reverse leakage, and the reverse pumping is realized.

#### 3.2.2. Dimple Depth

**Figure 12:**
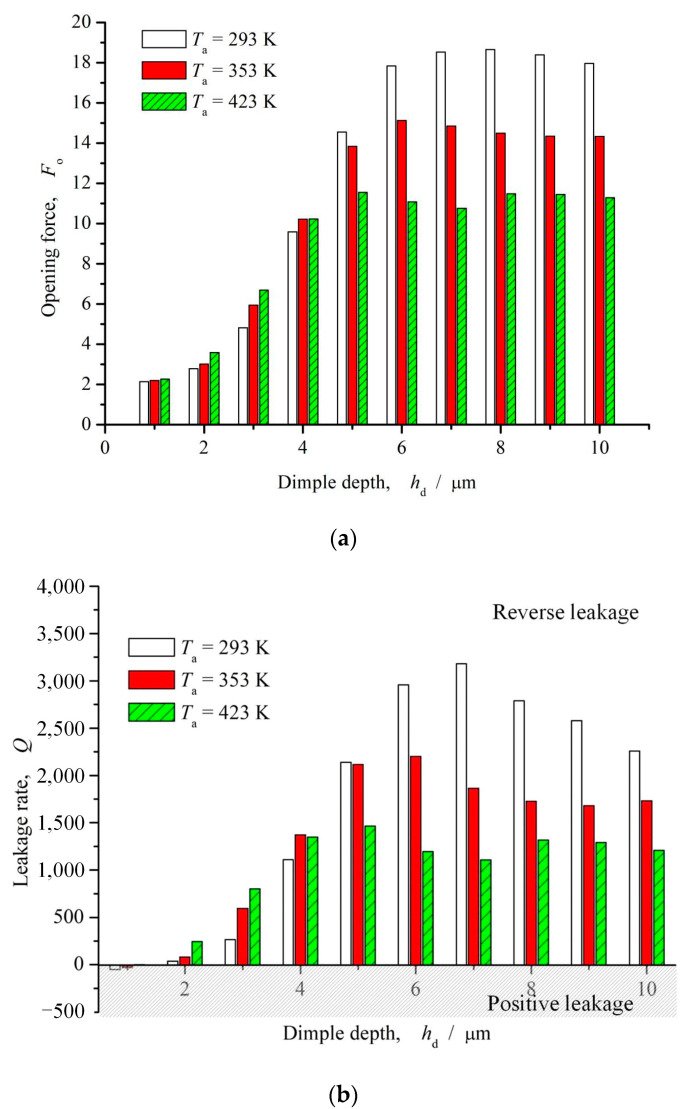
Influence of dimple depth on opening force and leakage rate (*ω* = 12,000 rpm, *P*_o_ = 4, *h*_0_ = 2 μm, *γ* = 3, *α* = 45°, *N* = 80). (**a**) Opening force; (**b**) Leakage rate.

Figure 12 shows the influence curve of dimple depth on opening force and leakage rate. As can be seen from the figure, with the increase of dimple depth, more fluid flows into the elliptical microdimples due to the further decrease of fluid flow resistance. Under the action of velocity shear, upstream pumping occurs, resulting in a gradual increase in opening force and a steady trend. The leakage gradually changes from forward pumping to reverse pumping with the increase of dimple depth and becomes stable when the dimple depth is greater than 8 μm. In addition, it can be seen that when the dimple depth is between 5 and 10 μm, the opening force is the largest and the reverse pumping effect is the strongest. The reason may be that, as the sealing temperature increases, the viscosity of the sealing medium gradually decreases. The lower the shear rate, the smaller the driving force of the rotational speed on the fluid, resulting in the lower the upstream pumping effect and opening force.

#### 3.2.3. Slender Ratio

**Figure 13:**
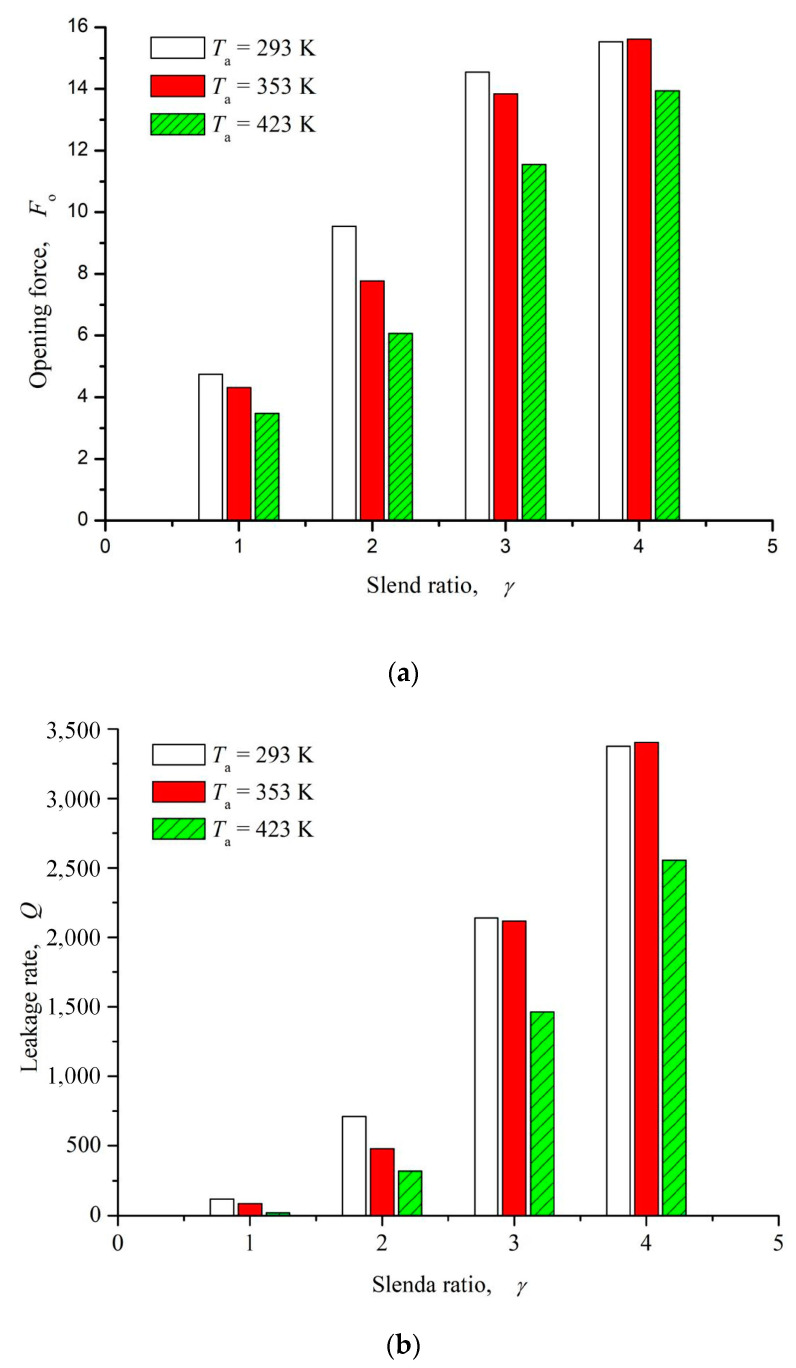
Influence of slender ratio on opening force and leakage rate (*ω* = 12,000 rpm, *P*_o_ = 4, *h*_0_ = 2 μm, *h*_d_ = 5 μm, *α* = 45°, *N* = 80). (**a**) Opening force. (**b**) Leakage rate.

Figure 13 shows the influence curve of slender ratio on opening force and leakage rate. As can be seen from the figure, with the increase of length-diameter ratio, the opening force presents a monotonically increasing trend, but when the length-diameter ratio *γ* > 3, the growth rate of opening force gradually decreases and tends to be stable. The changing trend of the leakage rate is consistent with the opening force, and presents the reverse leakage phenomenon. The main reason for this phenomenon may be that with the increase of slender ratio, the guiding effect of inclined elliptic microdimples on the fluid is enhanced, so the upstream pumping effect is strengthen, which results in enhancement of both hydrodynamic effect and reverse pumping effect.

## 4. Conclusions

In this study, a modified thermo-hydrodynamic lubricating model is established to investigate the leakage characteristics of upstream pumping face seal with inclined ellipse dimples. It can be found that the thermo-viscosity effect makes the cavitation intensity decrease obviously, leading to increase of upstream pumping effect of ellipse dimples, which play a positive role for the upstream pumping face seals. The values of both upstream pumping leakage rate and opening force may increase about 10%.

1. Under the condition of high-temperature and high-speed liquid lubrication, the inclined ellipse dimples can produce obvious upstream pumping effect, and zero leakage of the sealed medium can be achieved by increasing the dimple number and the slender ratio. Meanwhile, the hydrodynamic effect can make the opening force increase more than 50%.
2. The upstream pumping capacity of the seal face increases with the increase of the dimple depth, the number of circumferential dimples and the slender ratio. Here, when the dimple depth is 5–10 microns, the inclination angle is 45°, the number of circumferential dimples is greater than 30, and the slender ratio is greater than 3, the seal can realize the complete reverse pumping.
3. This study may provide the theoretical basis and design guidance for the upstreaming liquid face seals at high-temperature and high-speed conditions. In the future, the surface displacement of sealing ring can be coupling into the proposed model to make the simulation results more accuracy.

## Figures and Tables

**Table 1:** Structure parameters of the microporous texture.

Structure Parameters	Values
Textured radius *r*_g_/mm	10.5
Dimple number in radial directions *n*_r_	3
Dimple number in rotating directions *N*	80
Short axial diameter *b/*μm	200
Slender ratio *γ* = a/b	3
Dimple inclination angle *α/*°	45
Dimple depth *h*_d_/μm	5
Film thickness *h*_0_/μm	2

**Table 2:** Characteristics of the materials.

Characteristics	Carbon	Stainless Steel
Density/kg·m^−3^	1800	7800
Young’s modulus/GPa	25	204
Poisson’s coefficient	0.20	0.3
Specific heat capacity/J·kg^−1^·K^−1^	710	460
Thermal conductivity/W·m^−1^·K^−1^	15	16.4
Linear thermal expansion coefficient/10^−6^ K^−1^	4	15.9

**Table 3:** Parameters of lubricant.

Characteristics	Value
Initial viscosity *η*_0_	4 mPa·s
initial temperature *T*_0_	293 K
Temperature-viscosity coefficient *β*	0.031 K^−1^

## Data Availability

All data is contained within the article.

## References

[1] Etsion I. (2004). Improving tribological performance of mechanical components by laser surface texturing. Tribol. Lett..

[2] Wan Y., Xiong D.S. (2008). The effect of laser surface texturing on frictional performance of face seal. J. Mater. Process. Technol..

[3] Dingui K., Brunetière N., Bouyer J., Adjemout M. (2020). Surface texturing to reduce temperature in mechanical seals. Tribol. Online.

[4] Wang W., He Y.Y., Zhao J.Y., Mao J., Hu Y., Luo J. (2020). Optimization of groove texture profile to improve hydrodynamic lubrication performance: Theory and experiments. Friction.

[5] Yu H.W., Wang X.L., Zhou F. (2010). Geometric shape effect of surface texture on the generation of hydrodynamic pressure between conformal contacting surface. Tribol. Lett..

[6] Bai L.Q., Bai S.X. (2014). Frictional performance of a textured surface with elliptical dimples: Geometric and distribution effects. Tribol. Trans..

[7] Bai S.X., Song Y.S. (2019). Upstream pumping characteristic of inclined-ellipse-dimples on liquid-lubricated seal face. Tribology.

[8] Etsion I. (1984). A new concept of zero-leakage noncontacting mechanical face seal. J. Tribol..

[9] Akram S., Athar M., Saeed K., Razia A., Alghamdi M., Muhammad T. (2022). Impact of partial slip on double diffusion convection of sisko nanofluids in asymmetric channel with peristaltic propulsion and inclined magnetic field. Nanomaterials.

[10] Khan Y., Akram S., Razia A., Hussain A., Alsulaimani H.A. (2022). Effects of double diffusive convection and inclined magnetic field on the peristaltic flow of fourth grade nanofluids in a non-uniform channel. Nanomaterials.

[11] Payvar P., Salant R.F. (1992). A computational method for cavitation in a wavy mechanical seal. J. Tribol..

[12] Brunetière N., Rouillon M. (2021). Fluid flow regime transition in water lubricated spiral grooved face seals. Tribol. Int..

[13] Wang Q., Chen H.L., Liu T. (2012). Research on performance of upstream pumping mechanical seal with different deep spiral groove. Earth Environ. Sci..

[14] Xu L.S., Wu J.H., Wang Y.L., Li Z., Yuan X. (2020). Influence of squeezing behavior on cavitation characteristics and performance parameters of mechanical seals with an efficient mass-conserving algorithm. J. Braz. Soc. Mech. Sci. Eng..

[15] Lai T. (1994). Development of non-contacting, non-leaking spiral groove liquid face seals. Lubr. Eng..

[16] Lebeck A.O. (2008). Experiments and modeling of zero leakage backward pumping mechanical face seals. Tribol. Trans..

[17] Xie J., Bai S.X. (2017). Hydrodynamic characteristics of gas upstream pumping face seals texturing with inclined-ellipse-dimples. Tribology.

[18] Xie J., Bai S.X., Ma C.H. (2020). Hydrodynamic effect of non-closed elliptical grooves of bi-directional rotation gas face seals. Ind. Lubr. Tribol..

[19] Bai S.X., Hao J.L., Yang J., Song Y. (2022). Gas-liquid mass transfer behavior of upstream pumping mechanical face seals. Materials.

[20] Kou G.Y., Li X.H., Wang Y. (2020). Parameter study and shape optimisation of a generalised ellipse dimple-textured face seal. Lubr. Sci..

[21] Li Z., Hao M., Sun X., Yang C., Li Y., Wang Y. (2019). Experimental study of cavitation characteristic of single-row reverse spiral groove liquid-film seals. Tribol. Int..

[22] Zhang S., Jiang S., Lin X. (2020). Static and dynamic characteristics of high-speed water-lubricated spiral-groove thrust bearing considering cavitating and centrifugal effects. Tribol. Int..

[23] Liu D.H., Zhang B., Zhao J., Ge K., Zhang S. (2015). Analysis of a hydrodynamic spiral grooved upstream pumping face seal considering cavitation. Appl. Mech. Mater..

[24] Bai S.X., Wen S.Z. (2019). Gas Thermohydrodynamic Lubrication and Seals.

[25] Bai S.X., Song Y.S., Yang J. (2022). Elastic deformation of liquid spiral groove face seals operating at high speeds and low pressure. Int. J. Mech. Sci..

[26] Nau B.S. (1980). Observations and analysis of mechanical seal film characteristics. ASME J. Lubr. Technol..

[27] Li Z.T., Li Y.F., Cao H., Hao M., Liu F., Meng D. (2021). Investigation of cavitation evolution and hydrodynamic performances of oil film seal with spiral groove. Tribol. Int..

[28] Zhang J.Y., Meng Y.G. (2012). Direct observation of cavitation phenomenon and hydrodynamic lubrication analysis of textured surfaces. Tribol. Lett..

[29] Aggarwal S., Pandey R.K. (2017). Frictional and load-carrying behaviours of micro-textured sector shape pad thrust bearing incorporating the cavitation and thermal effects. Lubr. Sci..

[30] Zouzoulas V., Papadopoulos C.I. (2017). 3-D thermohydrodynamic analysis of textured, grooved, pocketed and hydrophobic pivoted-pad thrust bearings. Tribol. Int..

[31] Wu C.W., Zheng L.Q. (1989). An average reynolds equation for partial film lubrication with a contact factor. J. Tribol..

[32] Greenwood J.A., Tripp J.H. (1970). The contact of two nominally flat rough surfaces. Proc. Inst. Mech. Eng..

